# Macroplastic accumulation across different surface covers, a case study of two South African rivers

**DOI:** 10.1038/s41598-025-26494-z

**Published:** 2025-11-27

**Authors:** Thendo Mutshekwa, Musa C. Mlambo, Liliso Mbadla, Solethu Mkhencele, Lubabalo Mofu, Miracle O. Osoh, Samuel N. Motitsoe

**Affiliations:** 1 Department of Freshwater Invertebrates, Albany Museum, Makhanda, 6139 South Africa; 2 Institute for Water Research, Rhodes University, Makhanda (Grahamstown) 6140, South Africa; 3 School of Animal, Plant and Environmental Sciences, University of the Witwatersrand, Johannesburg, 2050 South Africa; 4 South African Institute for Aquatic Biodiversity, Makhanda, 6140 South Africa; 5 Department of Ichthyology and Fisheries Science, Rhodes University, Makhanda, 6139 South Africa

**Keywords:** Plastic-wood jams, Macroplastic storage, River pollution, Plastic debris, Physical pollution, River hydrodynamics, Ecology, Ecology, Environmental sciences

## Abstract

Plastic pollution in freshwater ecosystems is an escalating global concern, yet riverine macroplastic pollution and dynamics remain poorly understood in Southern Africa. This study investigated macroplastic accumulation across different surface covers in two rivers with contrasting anthropogenic inputs. We hypothesised that wood jams in the current study would contribute to high macroplastic loads across Bloukrans and Palmiet rivers and that the polymer composition of plastics would be diverse across all four surface covers. A total of 452 macroplastic items were collected, with significantly higher loads recorded in the Bloukrans River (3.5 items/m^2^; 93.3 g/m^2^) compared to the Palmiet River (0.8 items/m^2^; 10.1 g/m^2^). Multilayer plastics and soft polyolefins dominated across both rivers, particularly in herbaceous vegetation and wood jams. Significant differences were observed in macroplastic loads between rivers and among surface covers for Palmiet River. The increased macroplastic loads in the Bloukrans River were associated with untreated municipal effluent, stormwater runoff, and informal waste dumping, while in Palmiet River, macroplastic pollution was mainly linked to recreational littering and runoff. Wood jams emerged as an effective hotspot for macroplastic debris. These findings highlight the critical role of surface cover in enabling macroplastic accumulation and distribution thus the study further calls for targeted waste management, cleanup operations, and riparian protection strategies to safeguard river health and resilience in Southern Africa.

**Supplementary Information:**

The online version contains supplementary material available at 10.1038/s41598-025-26494-z.

## Introduction

Plastic pollution has emerged as a major global environmental concern, gaining prominence in ecological research due to its scale, persistence, and widespread impacts^1–3^. This is because, plastic production has risen sharply over the past fifty years, due to its affordability, versatility, and long-lasting/persistence in the environment^4,5^. However, a substantial portion of plastic waste is disposed in landfill sites where majority still escapes into the natural environment^6^, where it accumulates and persist for many years, posing long-term ecological and health risks^7,8^. In aquatic environments, plastics are of particular concern as they alter habitat structure, disrupt aquatic food webs, and threaten aquatic biodiversity^9–11^.

Historically, studies on plastic pollution were centred on marine ecosystems^12–14^, but there is a growing shift to focus on inland freshwater systems^11^, including rivers^15,16^, lakes^17^, and reservoirs^18^. Among these ecosystems, riverine systems have received increased attention since they not only serve as critical habitats for diverse aquatic life but also act as the primary pathways through which plastic debris is transported from terrestrial sources to marine environments^19,20^. Riverine systems play a pivotal role in the transport and accumulation of plastic debris due to their catchment drainage nature, one-way water flow including dynamic hydrological and geomorphological characteristics^21^. Factors such as flow velocity, channel morphology, vegetation cover, and the presence of natural or artificial barriers can influence the retention and redistribution of macroplastic items along a river continuum^22–24^. Slow-flowing sections, meanders, riparian vegetation, and wood jams often act as traps, where plastics accumulate for extended periods before being remobilized during high-flow events^22^. Moreover, rivers frequently receive direct inputs of plastic waste from densely populated urban areas and informal settlements and effluents from wastewater treatment plants, where inadequate domestic waste management and poor sanitation infrastructure exacerbate pollution loads^25^. Consequently, rivers function not only as pathways for plastic transport to lakes and oceans but also as major retention zones, where the build-up of plastic waste and associated pollutants may have serious implications for water quality^26^, biodiversity^27^, and public health^28^.

Several studies have investigated macroplastic accumulation in riverine systems^29,30^. However, few studies have investigated how different surface covers influence the distribution and accumulation of macroplastics^31,32^ and, to our knowledge, no such study has been conducted in Southern Africa rivers. To contribute to the baseline data on macroplastic pollution in riverine systems in Southern Africa, the present study was initiated to investigate how different surface cover types i.e., exposed sediments, herbaceous vegetation, woody vegetation, and wood jams influence macroplastic accumulation in two river systems with contrasting anthropogenic inputs in Southern Africa. In additionally, to assess the potential effect of macroplastic load on river water quality, water quality parameters were measured *in-situ* to correlate with macroplastic abundance and distribution. The terminologies of surface covers were adopted from Liro and Gallitelli^31^, who reported high macroplastic loads in wood jams. Thus, the present study hypothesise that wood jams would contribute to high macroplastic loads across the Bloukrans and Palmiet rivers and that the polymer composition of plastics would be diverse across all four surface covers (i.e. exposed sediments, herbaceous vegetation, woody vegetation, and wood jams). We further hypothesised that Bloukrans River, which is subjected to higher anthropogenic inputs, would exhibit a greater overall macroplastic debris load when compared to Palmiet River. Findings from this study provide important insights into the role of surface covers in mediating plastic retention and highlight the need to consider landscape features in designing effective monitoring and mitigation strategies for plastic pollution in riverine environments.

## Materials and methods

### Study area

**Fig. 1:**
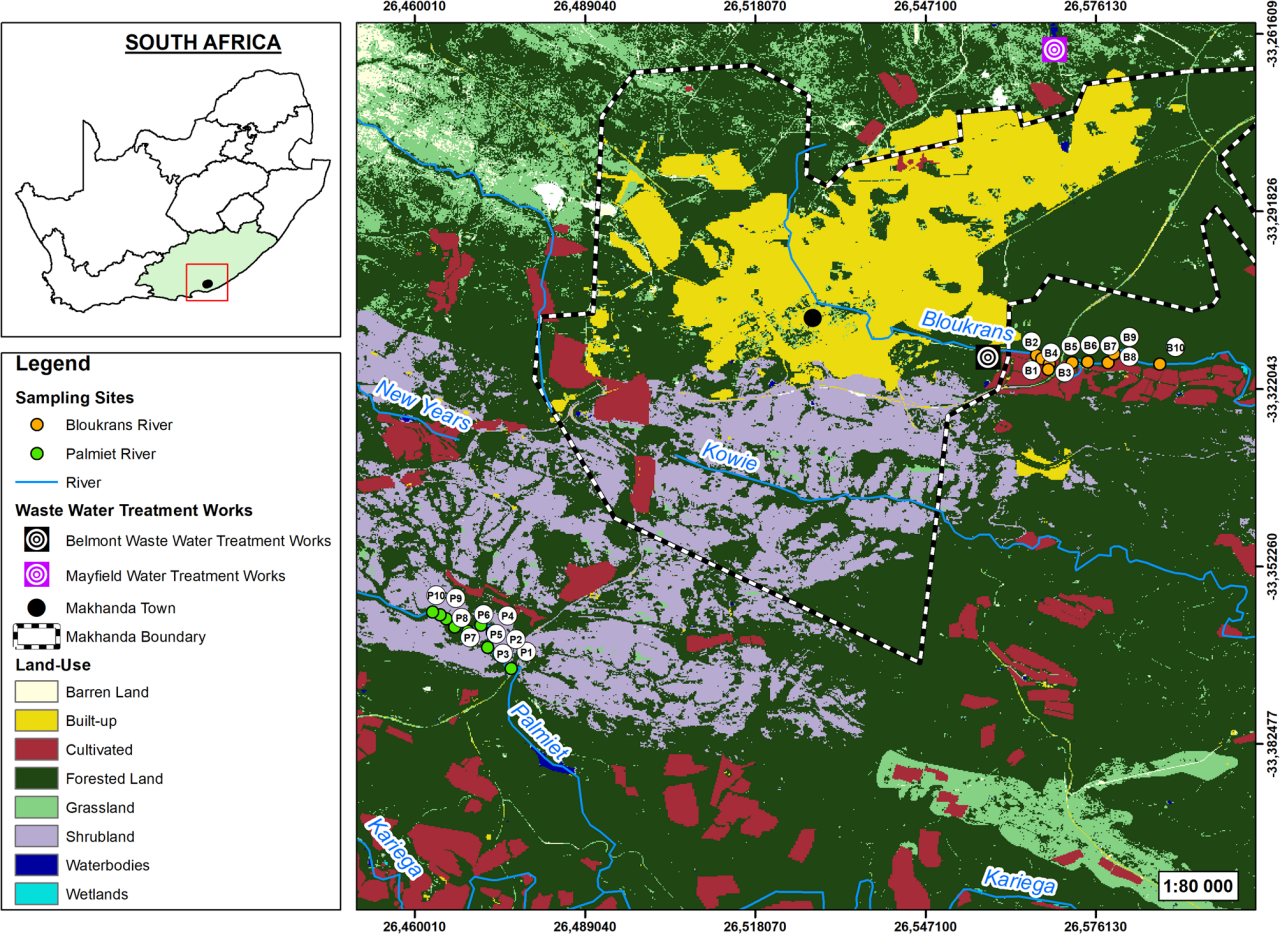
Map showing the study area, highlighting the Bloukrans and Palmiet Rivers in the Eastern Cape Province, South Africa. Image created using ArcGIS Desktop (version 10.8; https://www.esri.com/en-us/arcgis/products/arcgis-desktop/overview) with tools from the Data Management Toolbox (“*Calculate Field*”), and QGIS (version 3.28; https://qgis.org/), using the dataset “*MDB District Municipal Boundary 2018*” obtained from the Municipal Demarcation Board of South Africa (https://www.demarcation.org.za/).

The study was conducted along two river systems i.e., Bloukrans and Palmiet rivers, located in the Eastern Cape Province of South Africa (Fig. 1). These rivers flow through the semi-arid Albany region, which falls within the Grassland and Savanna biome transition zone which is characterized by a mosaic of urban, peri-urban, and rural land use^33^. The Bloukrans River drains the eastern side of Makhanda (formerly Grahamstown), passing through the densely populated urban setting and formal settlements (i.e. population ~ 70,000; https://worldpopulationreview.com/) (Fig. 1). The Bloukrans River is associated with higher levels of anthropogenic influence, particularly untreated wastewater discharges and stormwater inputs^33,34^. The river originates from the high-lying grasslands and rocky hills north of Makhanda and flows in a generally southeasterly direction before eventually joining the Kowie River which flows in the Indian ocean in Port Alfred town^33,35^. The Bloukrans River catchment area is approximately ~ 230 km^2^, with a total river length of ~ 59 km before the confluence with the Kowie River^36^.

The Palmiet River originates in the hills located southwest of Makhanda (Fig. 1). The river is a small tributary of the Kariega River, with a total river length of ~ 10.5 km and draining a catchment area of approximately 17 km^2^. Compared to the Bloukrans River, the Palmiet River is less disturbed, receiving impact mostly from recreational and cultural use such as hiking, camping and spiritual activities^34^. The river flows through closed hills, moderate and high relief within the Southern folded mountains ecoregion, dominated by a mosaic of grasslands, valley bushveld, semi-succulent woodland and savanna patches^37^. The mean annual rainfall across the two catchments ranges between 600 and 800 mm, mostly during spring and summer. Temperatures in the region range from a minimum of 1.5 °C in Winter to a maximum of ~ 40 °C in summer (South African Weather Services; https://www.weathersa.co.za/).

### Water quality parameters

During each sampling event, water quality parameters were measured at each site using a multi-parameter probe (Aquaprobe AP-800, AquaRead, England). These parameters included water temperature (°C), oxidation–reduction potential (ORP; mV), pH, electrical conductivity (EC; µS·cm⁻^1^), total dissolved solids (TDS; mg·L⁻^1^), and salinity (PSU).

### Macroplastic sampling

**Fig. 2:**
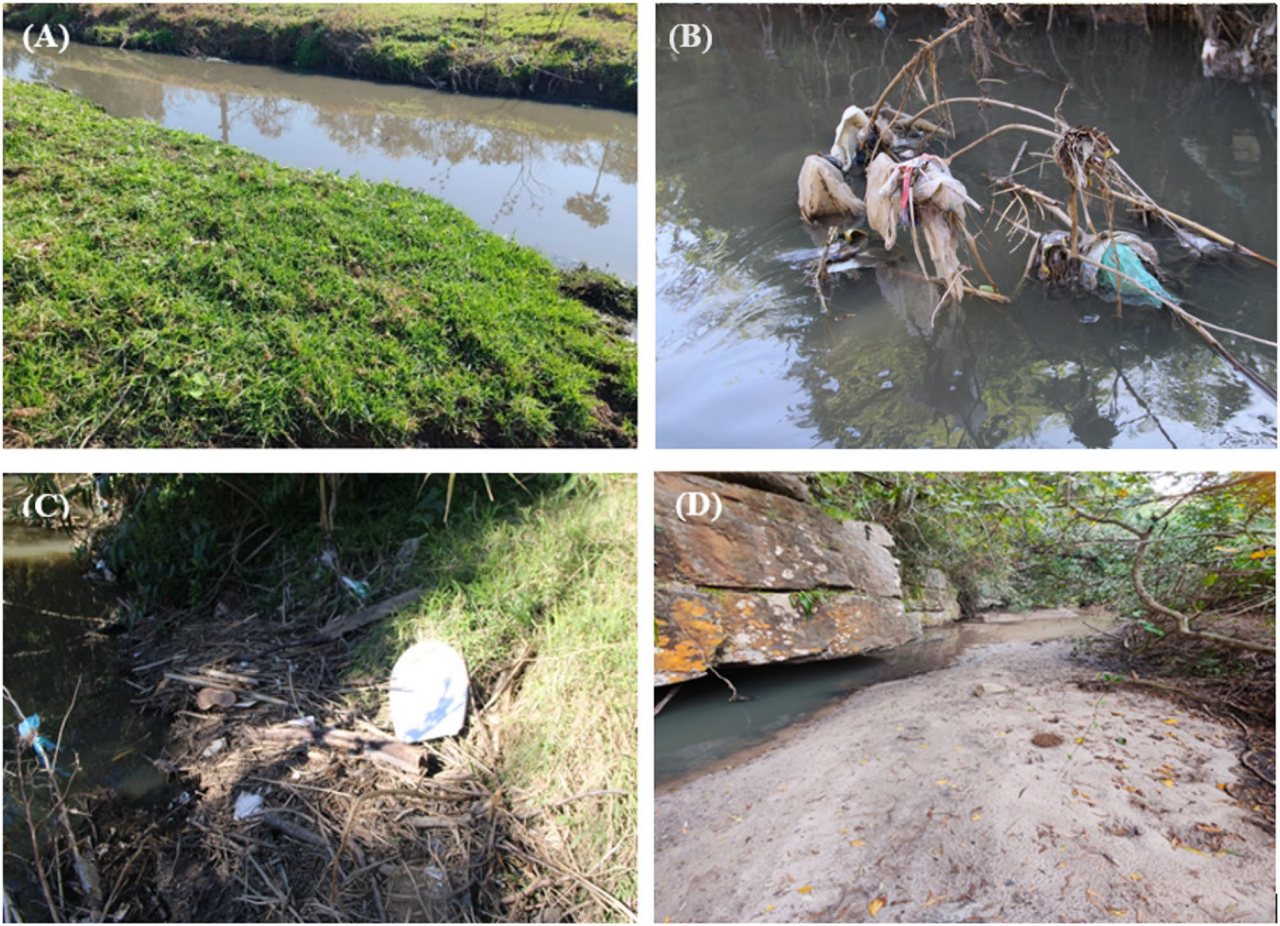
Example of different surface covers: (**A**) herbaceous vegetation, (**B**) wood jams, (**C**) woody vegetation in the Bloukrans River, and (**D**) exposed sediment in the Palmiet River (Photo by Thendo Mutshekwa).

Ten sites along a ~ 1.5 km stretch of the Bloukrans and Palmiet rivers where selected and sampled in July 2025 during dry-season, when low-flow conditions exposed the unvegetated gravel, sand, and mud biotopes along the river channel^38^. Following sampling technique adopted from Liro et al.^22^ and Liro and Gallitelli^31^’s studies, macroplastic items accumulated on four different types of physical surface covers i.e. exposed sediment, herbaceous vegetation, woody vegetation, and wood jams were collected (Fig. 2). Exposed sediment (Fig. 2D) were bare sandy or gravelly patches within the river channel or along its banks that lacked vegetation^22,31^. Herbaceous vegetation (Fig. 2A) comprised grasses and other non-woody plants along the riverbanks or within the channel that can intercept drifting plastics^22,31^. Woody vegetation (Fig. 2C) included shrubs and trees with living stems and roots along the riverbanks or within the channel, whereas wood jams (Fig. 2B) consisted of accumulations of dead woody debris lodged in the river channel^22,31^. Sites were only considered for sampling if they contained four or than two of these surface covers. All macroplastic items visible on each surface cover at each site were carefully hand-picked and placed into labelled polyethylene refuse bags (width 750 mm; 900 mm length) for laboratory analysis. The surface area of each surface cover was measured onsite (with wood jams measured across their entire extent), to enable the calculation of macroplastic item (items/m^2^) and mass (g/m^2^), with surface areas ranging from 0.3 to 3 m^2^.

### Sample processing

In the laboratory, collected macroplastic samples were left to air-dry for 24 h before processing to remove any adhering materials, such as sediment, sand, or organic matter. Once dried, samples were weighed and categorised into seven polymer groups per site and river system (Table S5); polyethylene terephthalate (PET: bottles), polystyrene (PS; cutlery, cups, plates), expanded polystyrene (EPS: foams, food containers), hard polyolefins (PO hard: bottle caps, containers, rigid items), soft polyolefins (PO soft: bags, films, sheeting), multilayer items (composite materials, food wrappings and packaging), and other plastics^31,39,40^. For macroplastic items (> 5 mm), polymer groups were assigned based on visual characteristics such as shape, colour, texture, and labelling, which is considered reliable for larger plastic items, as such spectroscopic techniques (e.g., FTIR or Raman) were not required.

### Statistical analysis

The Shapiro–Wilk test indicated non-normality. Consequently, the Kruskal–Wallis test was employed to compare differences in macroplastic items per square meter, average mass per square meter, and water quality parameters both within and between the two river systems. Statistical significance was inferred at *p* < 0.05^41^, and all analyses were conducted in R Ver 3.5. Principal component analysis (PCA) was applied to examine variations in macroplastic composition across different surface covers within each river using PAST Ver 4.03.

## Results

### Water quality parameters

Bloukrans River exhibited higher mean values for all measured water quality parameters including water temperature (12.12 ± 0.97 °C), ORP (150.4 ± 8.14 mV), pH (7.01 ± 0.05), EC (3165.5 ± 94.97 μS/cm), TDS (2063.3 ± 61.96 mg/L), and salinity (1.58 ± 0.11 PSU) (Table 1). In contrast, Palmiet River showed the lowest mean values for all water quality parameters: water temperature (11.34 ± 0.22 °C), ORP (106.1 ± 21.61 mV), pH (6.76 ± 0.08), EC (327.9 ± 107.51 μS/cm), TDS (213.6 ± 70.28 mg/L), and salinity (0.11 ± 0.04 PSU) (Table 1). Kruskal–Wallis test revealed statistically significant differences in all measured water quality parameters between Bloukrans River and Palmiet River (Table 1).

**Table 1:** Mean (± standard deviation) water chemistry variables measured across sampled rivers (Bloukrans River; Palmiet River) and Kruskal–Wallis results for each variable across the two rivers.

Parameter	Bloukrans River Mean ± SD	Palmiet River Mean ± SD	H-statistic	*p*-value
Water Temperature (°C)	12.25 ± 0.76	11.30 ± 0.23	14.31	**0.00016**
ORP (mV)	150.07 ± 7.68	109.62 ± 18.35	10.08	**0.00150**
pH	7.00 ± 0.04	6.73 ± 0.10	13.74	**0.00021**
EC (μ/cm)	2923.40 ± 607.98	364.10 ± 107.06	14.33	**0.00015**
TDS (mg/L)	1893.70 ± 393.51	236.90 ± 69.68	14.30	**0.00016**
Salinity (PSU)	1.45 ± 0.32	0.12 ± 0.03	14.91	**0.00011**

*ORP* oxidation–reduction potential, *EC* electrical conductivity, *TDS* total dissolved solids. Significant p-values are in bold.

#### Macroplastic loads

**Fig. 3:**
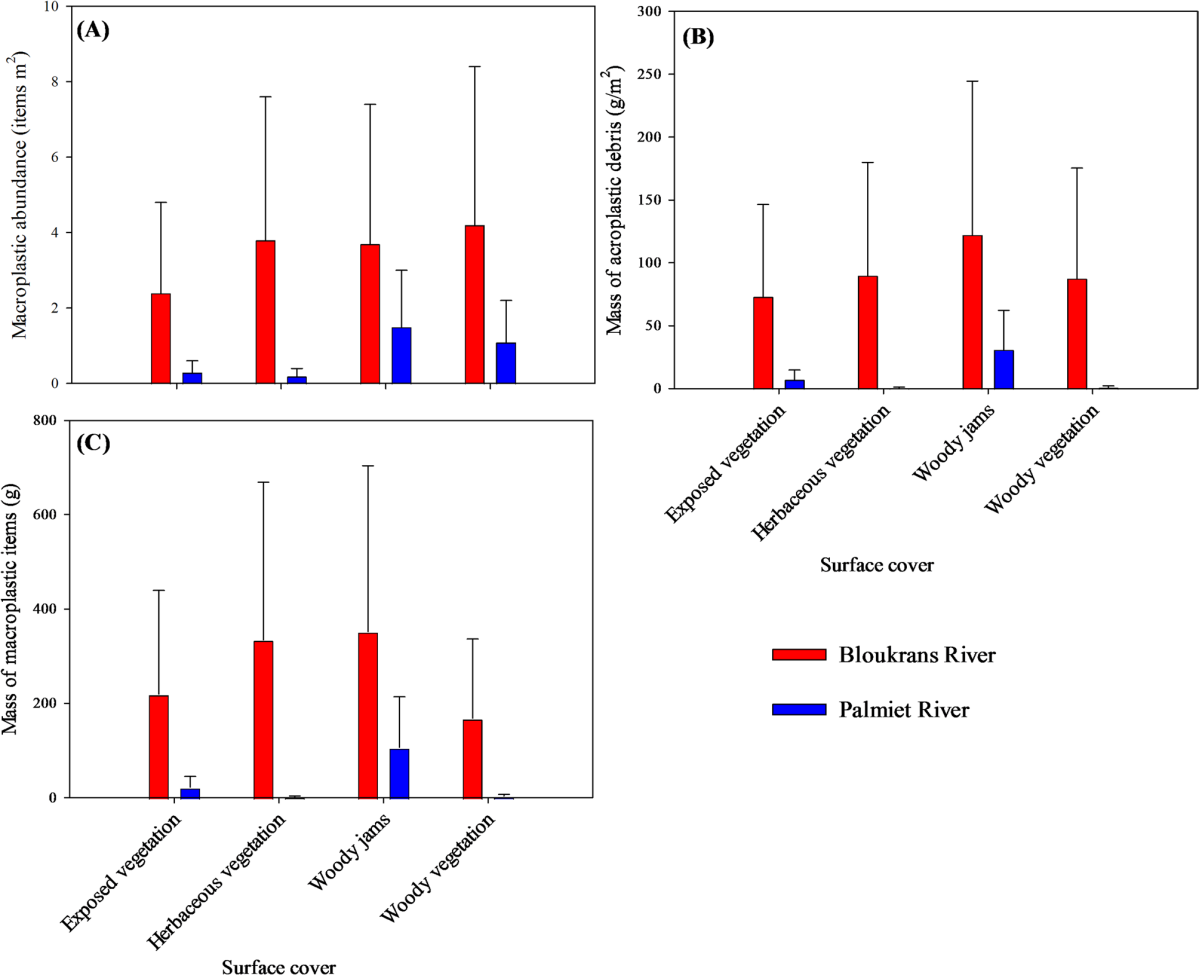
Mean (± SD) values for macroplastic (**A**) items per unit, (**B**) mass per unit, and (**C**) mass of macroplastic items across four surface covers: exposed sediments (Bloukrans River (*n* = 10); Palmiet River (*n* = 8)), herbaceous vegetation (Bloukrans River (*n* = 10); Palmiet River (*n* = 7)), woody vegetation (Bloukrans River (*n* = 10); Palmiet River (*n* = 8)), and wood jams (Bloukrans River (n = 9); Palmiet River (*n* = 8)), in Eastern Cape, South Africa.

A total of 452 macroplastic items were collected i.e. 401 in Bloukrans River and 51 Palmiet River across different surface covers. On average, macroplastic loads in the Bloukrans River ranged from 2.4 ± 0.6 to 4.2 ± 0.8 items/m^2^ (average: 3.5 items/m^2^) and 0.3 ± 0.1 to 1.5 ± 0.6 items/m^2^ (average: 0.8 items/m^2^) in Palmiet River (Fig. 3A; Table S1). Mass of macroplastics (g/m^2^) ranged from 73.2 ± 20.5 to 122.2 ± 33.1 g/m^2^ (average: 93.3 g/m^2^) and 0.6 ± 0.5 to 31.1 ± 8.2 g/m^2^ (average: 10.1 g/m2) in Bloukrans River and Palmiet River, respectively (Fig. 3B,C; Table S1). The higher mean values of water quality parameters in Bloukrans (Table 1) corresponded with higher macroplastic loads, and the opposite trend was also observed Palmiet River, were lower mean values was supported by low microplastic load, thus indicative of better water quality. According to Kruskal–Wallis test, no significant differences were observed in macroplastic items per unit (H = 3.462, *p* = 0.326) and mass per unit (H = 2.446, *p* = 0.485) for Bloukrans River between surface covers, whereas significant differences were observed in macroplastic items per unit (H = 9.649, *p* = 0.022) and mass per unit (H = 11.008, *p* = 0.012) for Palmiet River between surface covers. When comparing across the rivers, Kruskal–Wallis test revealed statistically significant differences in both macroplastic items and mass per unit between different surface covers (H = 28.03, *p* = 0.0002 for item; H = 25.28, *p* = 0.0007 for mass), with Post-hoc analysis presented in Table S2.

### Macroplastic composition

**Fig. 4:**
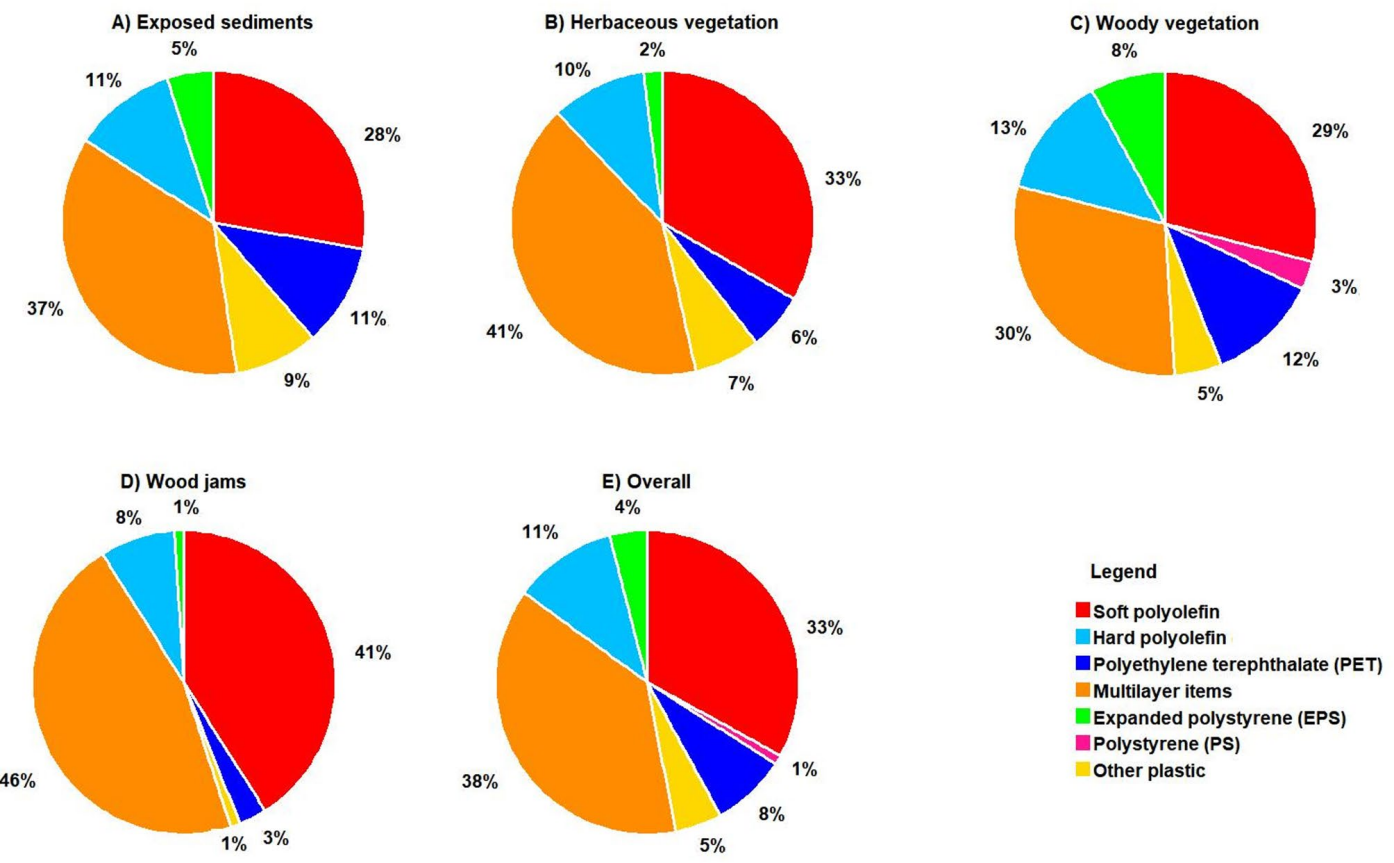
The overall percentage (%) distribution of polymer groups identified in Bloukrans River across various surface cover types: (**A**) Exposed sediments, (**B**) Herbaceous vegetation, (**C**) Woody vegetation, (**D**) Wood jams, and (**E**) Combined total.

**Fig. 5:**
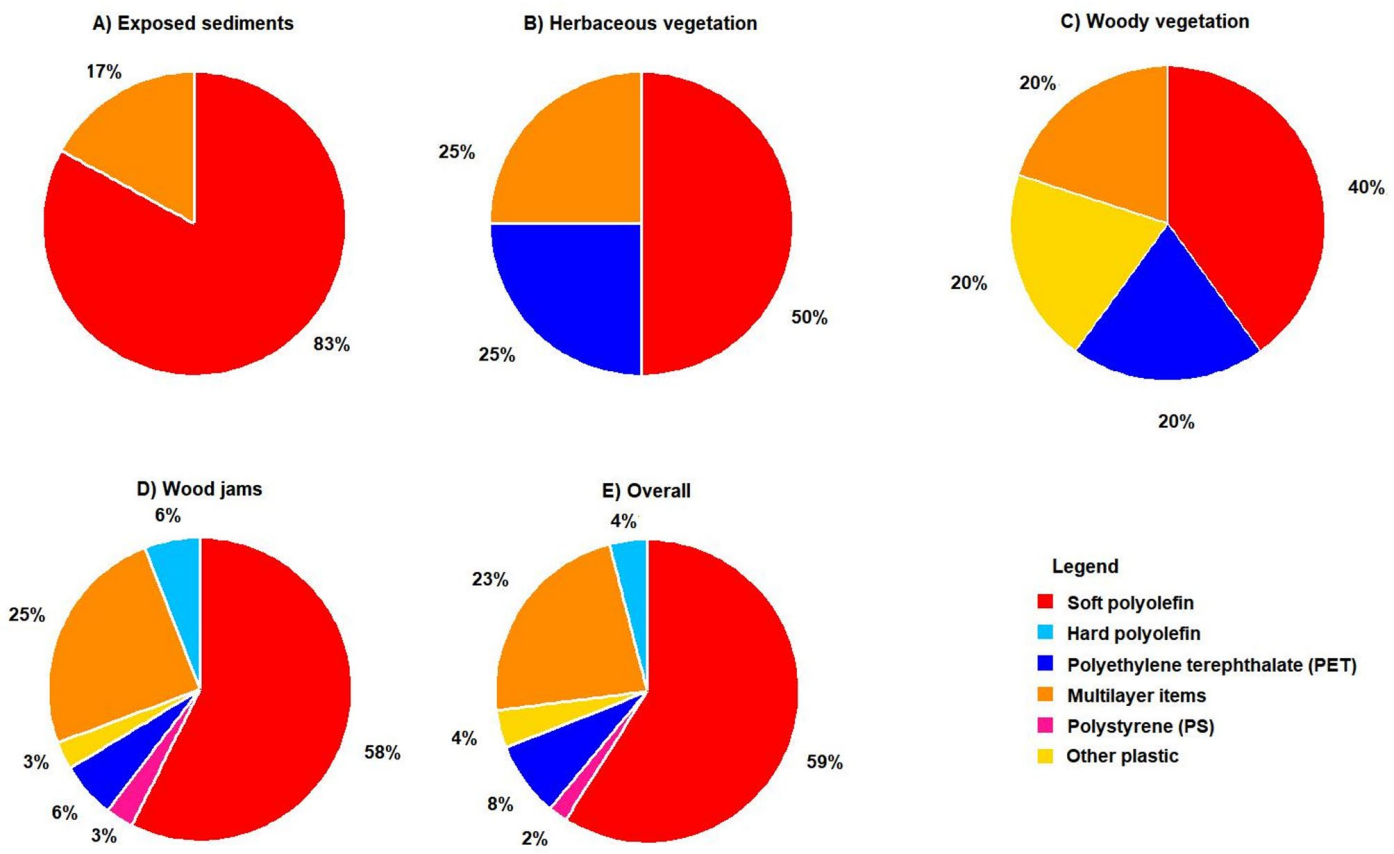
The overall percentage (%) distribution of polymer groups identified in Palmiet River across various surface cover types: (**A**) Exposed sediments, (**B**) Herbaceous vegetation, (**C**) Woody vegetation, (**D**) Wood jams, and (**E**) Combined total.

A variety of polymer groups were collected across the Bloukrans and Palmiet rivers (Figs. 4, 5). Among the collected polymer groups, the most common were multilayer items (e.g., food wrappings) (30–46%) and PO soft (e.g., foil bags) (28–41%), while PET (e.g., plastic bottles) (6–12%), PO hard (e.g., HDPE (High-Density Polyethylene) containers) (8–13%), EPS (e.g., foams) (1–8%), PS (e.g., cutlery) (0–3%), and others (e.g., cigarette butts) (1–9%) were less common in the Bloukrans River (Fig. 4). Specifically, multilayer items dominated in herbaceous vegetation (41%, Fig. 4B) and wood jams (46%, Fig. 4D), PO soft items were mostly found in wood jams (41%) and herbaceous vegetation (33%), and PET bottles were more frequent in woody vegetation (12%, Fig. 4C) and exposed vegetation (11%, Fig. 4A).

**Fig. 6:**
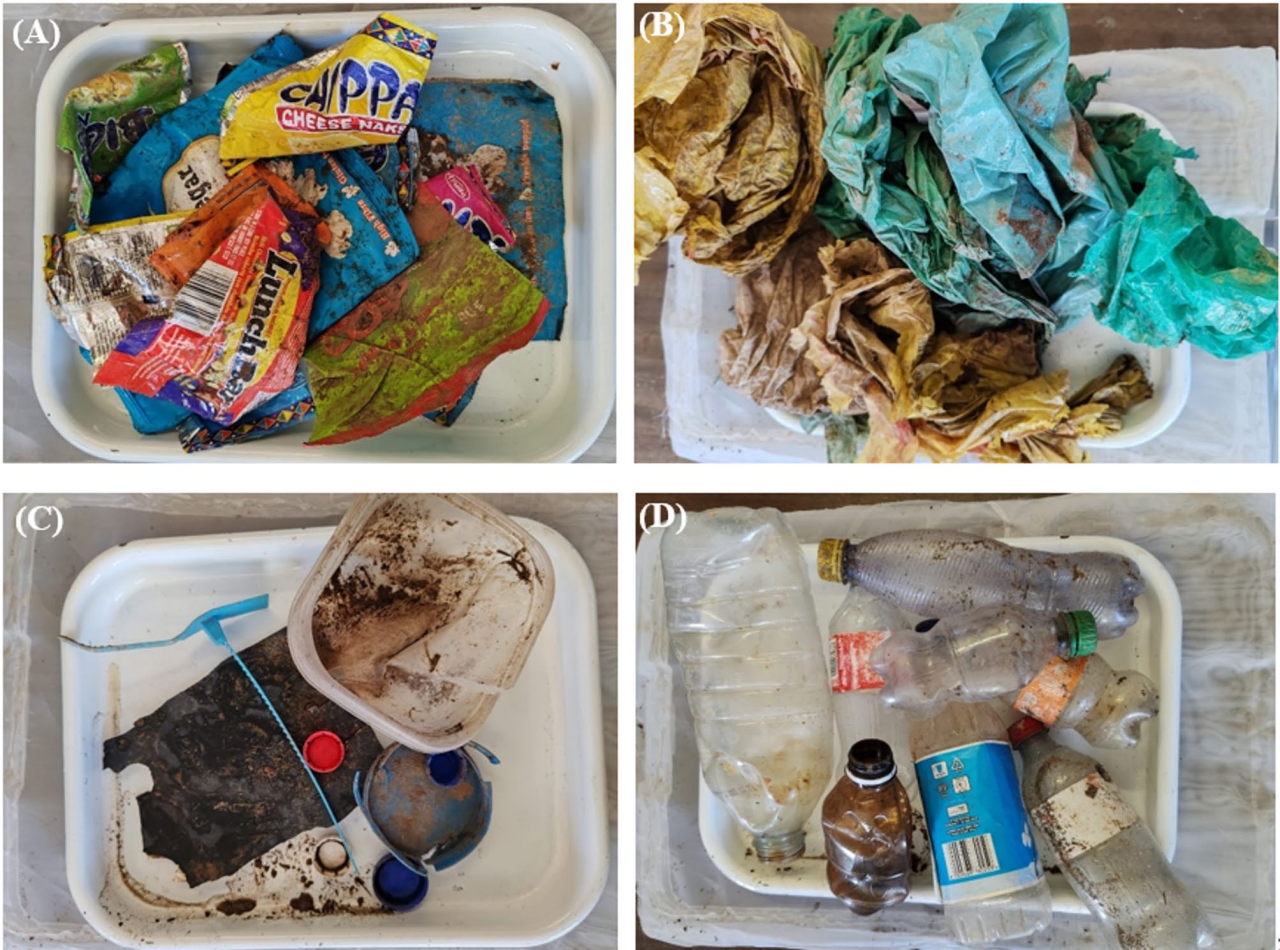
Examples of macroplastic items identified include: (**A**) Multilayer items (e.g., chip packets, sachets), (**B**) PO soft (e.g., plastic bags), (**C**) PO hard (e.g., bottle caps, containers, household items), and (**D**) PET plastics (e.g., beverage bottles) (Photo by Thendo Mutshekwa).

In Palmiet River, the most common macroplastic items were PO soft (e.g., foil bags) (40–83%), followed by multilayer items (e.g., wood wrappings) (17–25%), while PET (e.g., plastic bottles) (0–25%), PS (e.g., cutlery) (0–3%), PO hard (e.g., HDPE containers) (0–6%), and others (e.g., cigarette butts) (0–20%) were less common (Fig. 5). Specifically, PO soft items were dominant in exposed vegetation (83%, Fig. 5A) and wood jams (58%, Fig. 5D), multilayer plastics were frequent in wood jams (25%, Fig. 5D) and herbaceous vegetation (25%, Fig. 5B), and PET bottles were more common in herbaceous vegetation (25%, Fig. 5B) and woody vegetation (20%, Fig. 5C). Example of some different items were collected are shown in Fig. 6.

**Fig. 7:**
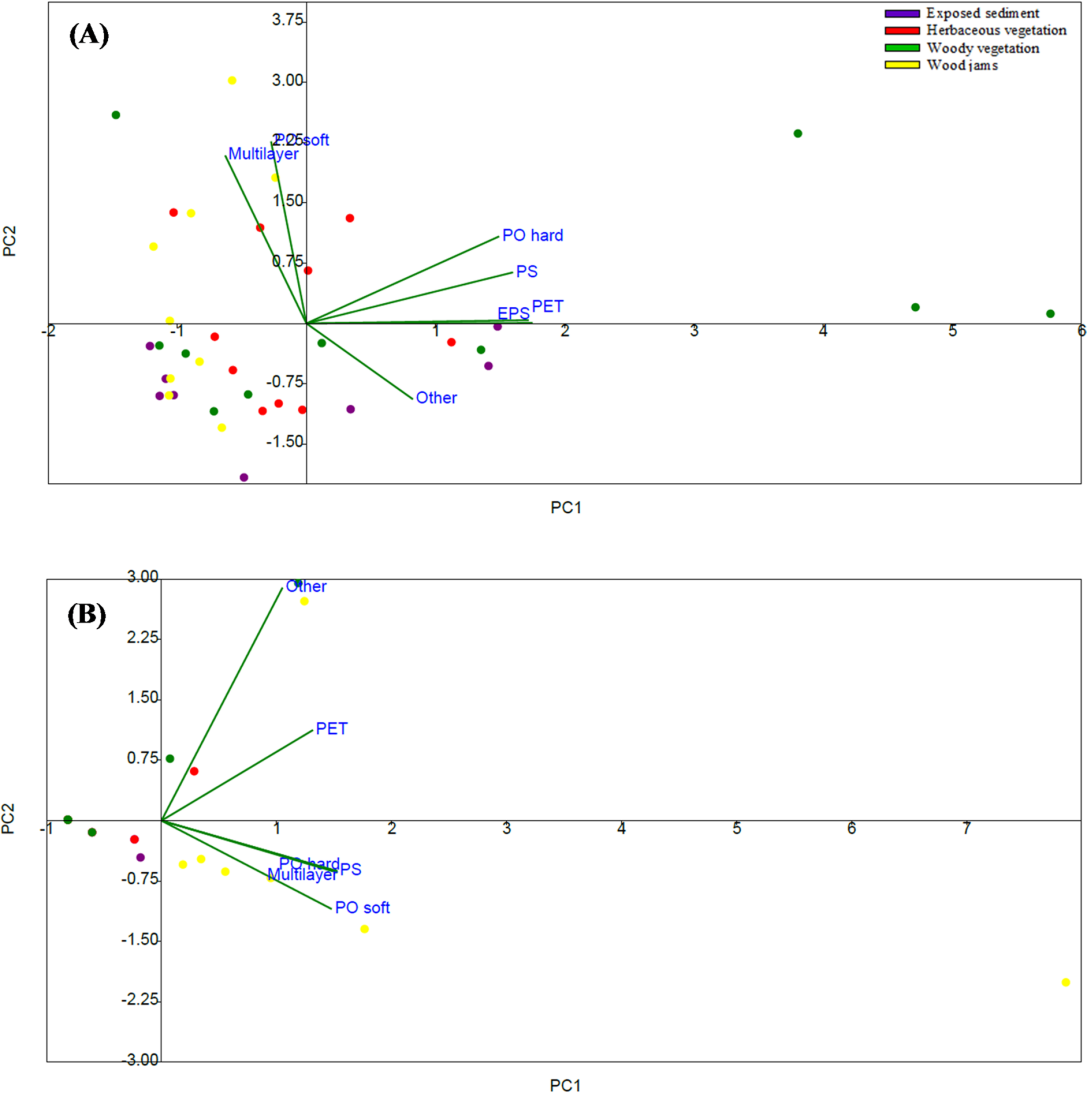
Principal component analysis (PCA) of macroplastic composition between surface covers within each study river i.e., (**A**) Boukrans River, (**B**) Palmiet River in the Eastern Cape, South Africa.

PCA results showed that axes 1 and 2 percentage variances was 55.73% (Eigenvalue 3.90) and 32.18% (Eigenvalue 2.25) respectively for the Bloukrans River, and 83.83% (Eigenvalue 5.03) and 11.45% (Eigenvalue 0.69) for the Palmiet River (Fig. 7; Table S3, S4). In Bloukrans River, wood jams were positively associated with Axis 1 with a strongly association with PO soft, Multilayer, PS, and PO hard (Fig. 7A). Exposed sediments and herbaceous vegetation cluster negatively to Axis 1, aligning more with “other” plastics and PET along Axis 2 (Fig. 7A). Woody vegetation is positioned near the origin, reflecting weaker association with specific plastic categories such as PET, PS, and multilayer plastics (Fig. 7A). In Palmiet River, wood jams were positively associated with Axis 1 and strongly associated with PO hard, Multilayer, and PS (Fig. 7B). Woody vegetation and exposed sediments cluster near the origin on Axis 2, with woody vegetation slightly positive and exposed sediments slightly negative (Fig. 7B). Herbaceous vegetation aligns negatively on Axis 2, thus closely associated with PO soft (Fig. 7B).

## Discussion

Macroplastic pollution is a worldwide environmental health issue, but research remains geographically uneven, with Southern African freshwater systems receiving little attention^42,43^. This study assessed macroplastic accumulation associated with different surface covers in two South African rivers with varying anthropogenic inputs and land use. In the Bloukrans River, wood jams did not retain the highest number of macroplastic items but did hold the greatest mass, whereas in the Palmiet River, wood jams captured both the highest item density and mass, making them the most effective macroplastic trap in the system. The hypothesis that wood jams would retain high macroplastic loads across both rivers was therefore only partially supported. Across all surface covers and in both rivers, multilayer plastics and soft polyolefins (PO soft) dominated, suggesting various sources of macroplastic inputs^22^. Also, PCA results showed that wood jams consistently acted as major accumulators of diverse plastics, whereas exposed sediments, herbaceous, and woody vegetation displayed weaker or more selective correlations. Water quality results revealed that Bloukrans River consistently exhibited poor water quality parameters when compared to Palmiet River, indicating greater exposure to anthropogenic inputs in addition to macroplastic dominance^36^.

Assessing macroplastic pollution in riverine systems is critical, as rivers are major conduits transporting land-based plastic waste into downstream aquatic ecosystems and eventually into marine environment^26,44^. In the present study, macroplastic loads were significantly higher in the Bloukrans River than in the Palmiet River. We speculate that this is because the Bloukrans River flows through the densely populated Makhanda town before draining the large agricultural and natural vegetation downstream. Untreated or partially treated municipal effluent, illegal solid waste dumping, stormwater runoff, and waste from informal settlements^33,45,46^ (Fig. S1, personal observation), this could substantially increase macroplastic load in the river and these inputs may eventually be transported to the marine environment, supporting Ryan et al.^47^’s study, who reported macroplastic debris on South African coastal beaches. In contrast, the Palmiet River which does not directly receive wastewater effluent nor drains the urban part of the Makhanda town^33^, demonstrated much lower macroplastic loads. This suggests that although urban inputs are lower in this river, recreational leisure and cultural activities such as hiking and camping still contributes to macroplastic pollution and retention^48^ (Fig. S2, personal observation). Our findings are consistent with studies linking town/cities proximity to increased plastic inputs in riverine systems^49,50^. Few studies have examined macroplastic accumulation across similar surface covers as in the present study. For example, Liro and Gallitelli^31^ reported that wood jams in the Aniene River downstream of Trevi nel Lazio retained 1.4 items/m^2^ (81.1 g/m^2^), which is lower than the values observed in the Bloukrans River (3.5 items/m^2^; 93.3 g/m^2^) but higher than the Palmiet River (0.8 items/m^2^; 10.1 g/m^2^), highlighting the influence of hydrological and catchment-specific factors. Similarly, wood jams and wooded islands in the Dunajec River have been identified as significant macroplastic traps^22^, while riparian vegetation in central Italian rivers has also been shown to capture substantial amounts of macroplastics^51^. These studies support our findings that both herbaceous and woody vegetation can serve as important sinks for plastic debris. Collectively, our results emphasize the need for targeted interventions in urban-influenced rivers like the Bloukrans, where multiple sources contribute to macroplastic pollution.

Consistent with previous research, our findings reinforce that large quantities of debris can accumulate in riparian zones and river channels, with wood jams emerging as particularly effective retention for macroplastic^23,51^. Hydrological processes such as streamflow, rainfall patterns, and seasonal floods influence the mobilisation of macroplastics from exposed sediments and herbaceous vegetation into the main channel, where they are intercepted by woody structures^21^. Exposed vegetation, due to its direct exposure to overland flow during rain events, can rapidly release macroplastics into the river, while herbaceous vegetation may act as a partial filter, retaining debris temporarily before high flows which further transport macroplastic downstream^52^. Within the channel, large wood jams create low-velocity zones and complex structural barriers that efficiently retain both buoyant and submerged macroplastics^24,53^

The identification of plastic polymer types is critical for tracing the potential sources of pollution and determining whether they originate from the breakdown of macroplastic items linked to industrial, agricultural, urban settings or recreational activities^54,55^. In both the Bloukrans and Palmiet rivers, macroplastic composition was dominated by multilayer and PO soft plastics, though their distribution across surface covers varied. Multilayer and PO soft plastics were especially common in wood jams and herbaceous vegetation. In the Bloukrans River, which drains the urban centre of Makhanda and surrounding agricultural lands, we speculate that different polymer groups may have originated from various sources, including municipal wastewater treatment works or raw waste water spillage, urban runoff, informal dumping, and agricultural activities that introduce packaging waste (e.g., multilayer food wrappers) and plastic bag fragments (PO soft) into the river system^44,53^. In the Palmiet River, similar polymer groups were recorded, but we speculate that their predominance is likely a result of direct human littering along riverbanks and recreational areas such hiking and camping, where discarded plastic bags, films, and multilayer wrappers become entangled in riparian vegetation and wood jams^28,56^. Our findings align with those of Liro et al.^23^, Gallitelli et al.^51^, and Liro et al.^57^, who also reported a variety of polymer types across similar surface covers, consistent with the patterns observed in the present study.

The accumulation of macroplastics in riverine systems has been reported to have several implications for aquatic ecosystems^10,27,58^ and human health^28,59^. Plastics in aquatic environments can leach chemical additives (e.g., phthalates, flame retardants, bisphenols) and degradation byproducts, which are harmful to aquatic organisms and can bioaccumulate through food webs, posing potential risks to humans who consume contaminated fish and other aquatic resources^44,60^. Over time, macroplastics break down into microplastics^61–64^ and nanoplastics^65^ through physical abrasion, UV radiation, and microbial activity, further exacerbating pollution loads and increasing their bioavailability to aquatic biota^66^. This degradation process is of particular concern in rivers such as the Bloukrans and Palmiet, where plastics are accumulated for extended periods in low-flow zones, increasing the likelihood of fragmentation and the release of toxic compounds^67^.

Our findings highlight the pressing need for targeted interventions in urban-adjacent rivers, such as the Bloukrans and elsewhere in Southern Africa, where poorly managed physical waste, failing sewage infrastructure, and agricultural runoff create persistent macroplastic inputs. For less urbanised rivers such as the Palmiet, the prevention of recreational littering and illegal dumping should be prioritised to maintain current lower macroplastic loads. Given the role of hydrological features in trapping and redistributing plastics, future studies should quantify retention times in different surface covers, assess seasonal variability in trapping efficiency, and evaluate the downstream transport potential during extreme weather events. Such work will improve predictive models of plastic fate in freshwater systems, inform riparian vegetation management, and guide the design of in-stream litter interception structure.

## Conclusion

This study demonstrates that macroplastic pollution is prevalent in Southern African freshwater systems and that different surface cover plays a critical role in retaining plastic debris. Generally, wood jams were particularly effective, capturing the greatest mass of macroplastics, while herbaceous and woody riparian vegetation acted as hotspots for debris along river shorelines. The Bloukrans River, influenced by urban, formal and informal settlements inputs from Makhanda, exhibited higher macroplastic loads compared to the less urbanised Palmiet River, highlighting the impact of anthropogenic activities on riverine plastic pollution. Multilayer plastics and soft polyolefins dominated across both rivers, suggesting multiple pollution source across the two rivers. Our findings emphasize the need for integrated management strategies that combine improved municipal waste management, regulation of recreational littering, protection of riparian vegetation, and public awareness campaigns to reduce plastic inputs. In addition, targeted cleanup operations along riparian zones before debris enters river channels can prevent macroplastics from reaching the river and being trapped by wood jams, thereby mitigating downstream fragmentation, chemical release, and ecological contamination. Future research should focus on seasonal variability, retention times, and downstream transport to better predict plastic fate and inform effective riverine plastic mitigation strategies.

## Supplementary Information

Below is the link to the electronic supplementary material.

Supplementary Material 1

## Data Availability

Data generated or analysed during this study are available from the corresponding author upon reasonable request.
