# Supplementary material for: Macroplastic accumulation across different surface covers, a case study of two South African rivers
Source: Sci Rep. 2025 Nov 27;15:42410. doi: 10.1038/s41598-025-26494-z (PMC12661026; doi:10.1038/s41598-025-26494-z)
Supplement: Supplementary file 1 — Supplementary Material 1 [file 41598_2025_26494_MOESM1_ESM.docx]

Supplementary file

**Macroplastic accumulation across different surface covers, a case study of two South African rivers**

Thendo Mutshekwa et al.

*Corresponding author(s)

E-mail: thendomutshekwa@gmail.com (TM)

| Surface covers | item/m^2^ | g/m^2^ | gram |
| --- | --- | --- | --- |
| Bloukrans River | | | |
| Exposed Sediment (*n* = 10) | 2.4±0.6 | 73.2±20.5 | 219.6±61.4 |
| Herbaceous vegetation (*n* = 10) | 3.8±0.6 | 90±22.2 | 334.5±66.5 |
| Woody jam (*n* = 9) | 3.7±0.8 | 122.3±33.1 | 352±99.4 |
| Woody vegetation (*n* = 10) | 4.2±0.8 | 87.7±25.2 | 168.1±75.7 |
|  |  |  |  |
| Palmiet River | | | |
| Exposed Sediment (*n* = 8) | 0.3±0.1 | 7.4±3.9 | 22.3±11.6 |
| Herbaceous vegetation (*n* = 7) | 0.2±0.1 | 0.6±0.5 | 1.6±1.4 |
| Woody jam (*n* = 8) | 1.5±0.6 | 31.1±8.2 | 106.8±22.4 |
| Woody vegetation (*n* = 8) | 1.1±0.1 | 1.1±0.8 | 3.3±2.5 |

Table S1. Mean (± SD) values of macroplastic load found in the Bloukrans River and Palmiet River across different surface covers.

| Maroplastic loads | Post-hoc results | *p* |
| --- | --- | --- |
|  | **Post-hoc across Palmiet River** |  |
| items/m^2^ | Woody vegetation *vs* Wood jams | 0.008 |
|  | Herbaceous vegetation *vs* Wood jams | 0.012 |
|  | Exposed sediment *vs* wood jam | 0.019 |
| gram/m^2^ | Herbaceous vegetation vs Wood jam | 0.005 |
|  | Woody vegetation *vs* wood jam | 0.004 |
|  | Exposed sediment *vs* Wood jam | 0.034 |
|  | **Post-hoc across Rivers** |  |
| items/m^2^ | *Bloukrans River* - Exposed sediments *vs Palmiet River* - Exposed sediments: | 0.007 |
|  | *Bloukrans River* - Herbaceous vegetation *vs* *Palmiet River* - Exposed sediments: | 0.007 |
|  | *Bloukrans River* - Woody vegetation *vs Palmiet River* - Exposed sediments | 0.008 |
| gram/m^2^ | *Bloukrans River* - Exposed sediments *vs* *Palmiet River* - Exposed sediments | 0.016 |
|  | *Bloukrans River* - Woody vegetation *vs* *Palmiet River* - Sediment with vegetation | 0.007 |
|  | *Bloukrans River* - Sediment with vegetation *vs Palmiet River* - Woody vegetation | 0.009 |

Table S2. Post-hoc results following Kruskal-Wallis test for macroplastic item per square meter (Items/m^2^) and macroplastic per square meter (gram/m^2^).

Table S3: Principle component analysis (PCA) factor loadings for macroplastic composition across surface covers for Bloukrans River.

| Bloukrans River |  |  |
| --- | --- | --- |
|  | PC1 | PC2 |
| Eigenvalue | 3.90116 | 2.25284 |
| Variance (%) | 55.731 | 32.183 |
| Macroplastic composition |  |  |
| PO soft | -0.07911 | 0.10759 |
| PO hard | 0.43145 | 0.25029 |
| PET | 0.50624 | -0.016482 |
| Multilayer | -0.18178 | 0.2401 |
| EPS | 0.49757 | -0.20002 |
| PS | 0.46248 | -0.3254 |
| Other | 0.23835 | 0.84969 |

Table S4: Principle component analysis (PCA) results for macroplastic composition across surface covers for Palmiet River.

| Palmiet River |  |  |
| --- | --- | --- |
|  | PC1 | PC2 |
| Eigenvalue | 5.02973 | 0.686991 |
| Variance | 83.829 | 11.45 |
| Macroplastic composition |  |  |
| PO soft | 0.42636 | 0.24495 |
| PO hard | 0.44056 | 0.068822 |
| PET | 0.37958 | -0.84679 |
| Multilayer | 0.44048 | 0.037971 |
| PS | 0.44056 | 0.068822 |
| Other | 0.30321 | 0.46047 |

Table S5: Table used for classification of macroplastic items and polymer groups in the current study, following a study by Liro and Gallitelli (2025; <https://doi.org/10.1007/s10750-025-05875-5>)

| ^Type^ | ^Description^ | ^Polymer groups^ |
| --- | --- | --- |
| ^Soft^ | ^Plastic bags, wraps, packaging films^ | ^LDPE (Low-Density Polyethylene), HDPE^ |
| ^Hard^ | ^Bottlecaps, containers, household items^ | ^PP (Polypropylene), HDPE, PVC^ |
| ^PET bottles^ | ^Drink bottles (transparent)^ | ^PET (Polyethylene Terephthalate)^ |
| ^Multilayer^ | ^Chip packets, sachets (multiple plastic layers or plastic+metal)^ | ^PET/Alu/PE composites, PP-based^ |
| ^EPS^ | ^Polystyrene foam, takeaway containers^ | ^EPS (Expanded Polystyrene)^ |
| ^PS^ | ^Rigid polystyrene products (e.g., utensils)^ | ^PS (Polystyrene)^ |
| ^Other^ | ^Unidentifiable plastics or mixed/unknown^ | ^Various: could be PVC, PC, PA, etc.^ |


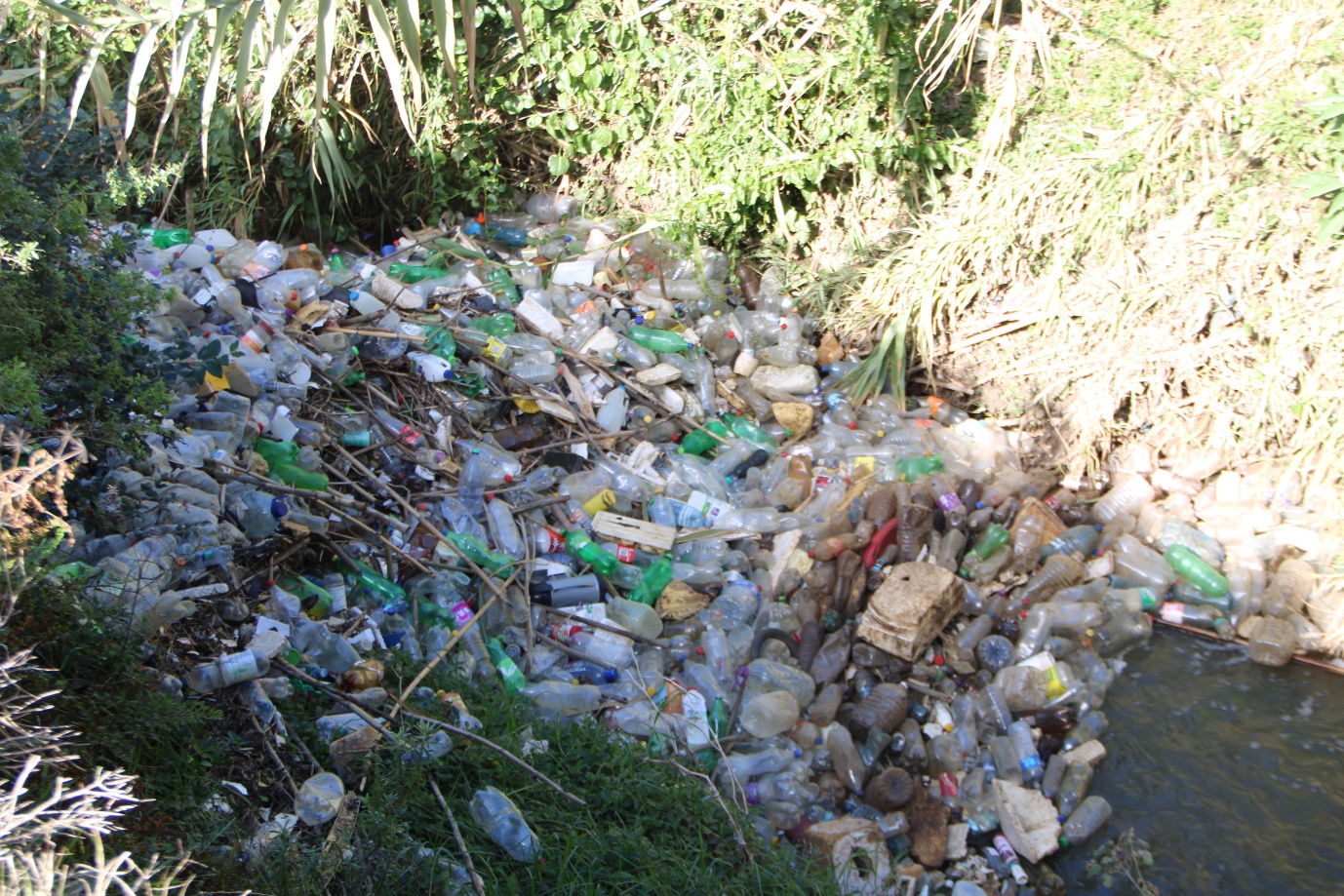


Figure S1: Plastic debris originating from untreated or partially treated municipal effluent, illegal solid waste dumping, stormwater runoff, and waste from informal settlements in Makhanda town, discharged downstream into the Bloukrans River (Photo by Thendo Mutshekwa).


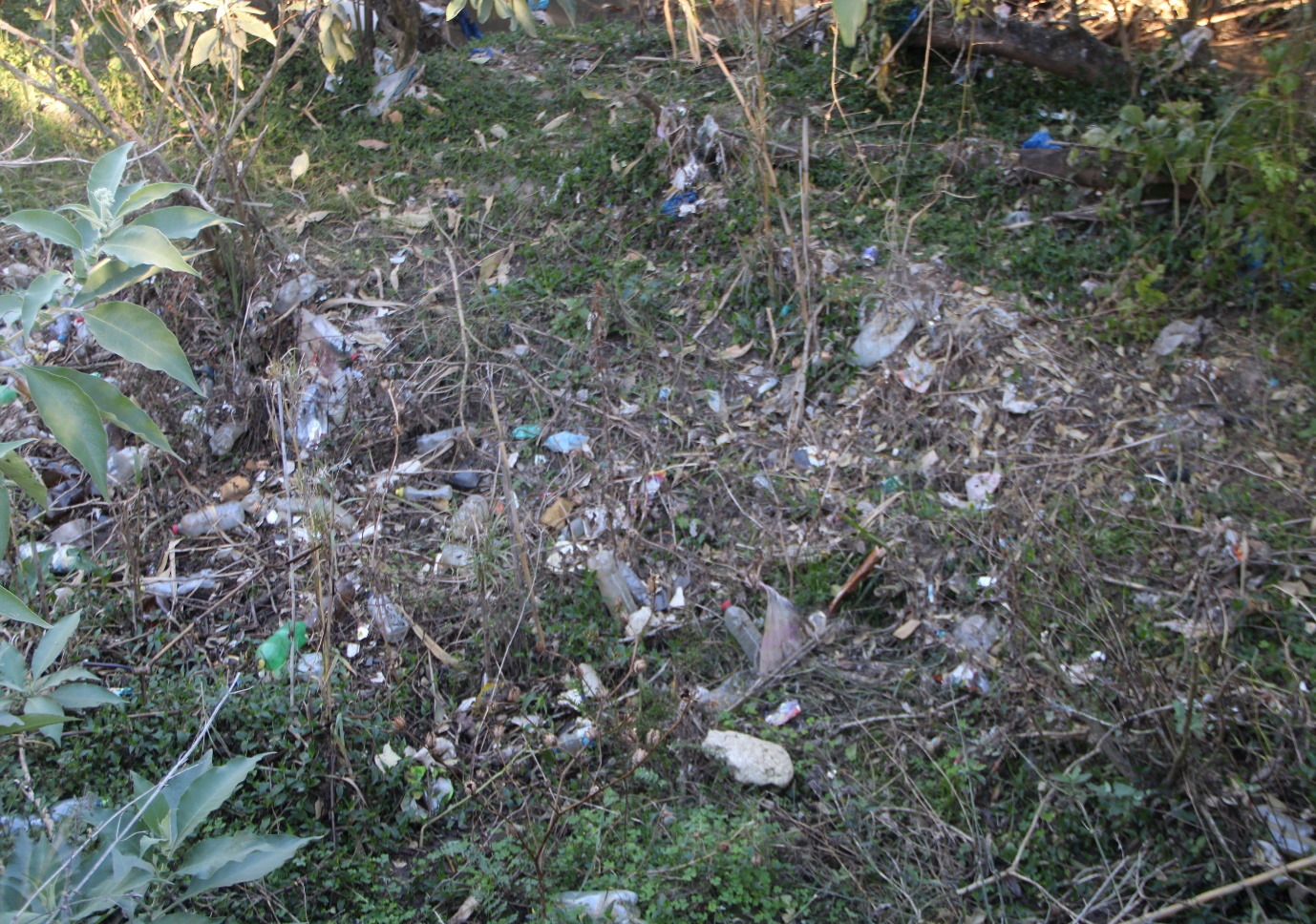


Figure S2: Illegal littering near the Palmiet River (Photo by Thendo Mutshekwa).
